# Constituent Parameter Identification of Braided Composite Based on Sensitivity Analysis

**DOI:** 10.3390/ma15248794

**Published:** 2022-12-09

**Authors:** Dong Jiang, Shitao Xie, Furong Qin, Dahai Zhang, Rui Zhu

**Affiliations:** 1 School of Mechanical and Electronic Engineering, Nanjing Forestry University, Nanjing 210037, China; 2 Institute of Aerospace Machinery and Dynamics, Southeast University, Nanjing 210096, China; 3 Institute of Flight System Dynamics, Technical University of Munich, 85748 Munich, Germany

**Keywords:** braided composite, inverse methods, constituent parameter identification, sensitivity analysis, response selection

## Abstract

Mechanical properties of the constituent material of fiber-reinforced braided composites will inevitably change after the manufacturing process. An approach to constituent parameters’ identification of braided composites was proposed to obtain the basic information of composites for structural analysis. Identification of the constituent parameters was transformed as an optimization problem, which was solved by adopting the sensitivity analysis method, iteratively minimizing the discrepancies between the numerically calculated displacement field and the measured displacement field. The sensitivity matrix of displacements with respect to the constituent parameters was directly derived based on the constitutive material model for the first time. Considering that the large magnitude differences between parameters will lead to an ill-posed problem of the sensitivity matrix, the identification was susceptible to noise from the experimental data, the relative sensitivity was adopted, and a condition number-based response point selection was applied to improve the robustness of the parameter identification. A 2.5-dimensional braided composite was employed to illustrate the constituent parameter identification method by comparing with the finite difference method. In addition, the influence of selected measuring points and measuring errors on the proposed method were discussed. The results showed that the proposed method can be used to identify the constituent parameters efficiently and accurately. When the measured displacements are polluted by noise, the condition number of the sensitivity matrix is an effective indicator of preceding information to enhance the identification accuracy.

## 1. Introduction

Braided composite materials have been widely applied in the aeronautics and aerospace industries due to their excellent mechanical properties. The knowledge of the microstructure and its mechanical properties provides essential information for composite structural design to satisfy the requirements of the macroscopic mechanical performance in different conditions. The manufacturing process of a composite, such as chemical vapor infiltration [1], thermal treatment [2], and pressure forming [3], can improve the macro-mechanical property but the constituent property of a composite will change after fabricating. Analyzing the macroscopic mechanical behavior of composite structures greatly depends on the mechanical properties of the fiber and the matrix [4,5,6] and the bonding conditions between constituents [7]. Constituent parameters after manufacture should be predicted prior to mechanical analysis and stimulation of composite structures [8].

The inverse method is widely applied to composite parameter prediction because it is difficult to directly measure [9]. Based on minimizing the cost function of a response residual between the analytical and experimental data, the composite parameters can be identified using the iterative optimization method. The construction of a response residual can use static displacement [10,11,12,13], strain data [14,15,16,17], and vibration responses [18,19,20,21]. A function is expressed as the discrepancies between the internal and external virtual work, corresponding to the virtual fields method (VFM) [22]. The advantage of the VFM is the superior computation efficiency; on the contrary, the disadvantage is the uncertainty of the selection of the virtual fields. Investigations on the identification of macroscopic composite material have attracted much attention. Although several non-gradient-based algorithms, such as genetic algorithms [23] and neural networks [24] can identify parameters with a data search, the superiority of gradient evaluations during identification is non-negligible. In general, this non-gradient-based optimization requires more than the many function evaluations of the gradient-based algorithm [25]. Common gradient-based identification approaches are developed based on the sensitivity analysis of a structural response with respect to constitutive structural parameters. For example, Huang [26] identified the elastic orthotropic parameters of functionally graded structures by combining the Levenberg–Marquardt method, in which the sensitivity calculation is based on the differentiation of the governing equations of the structural finite element method. Charkas et al. [15] calibrated a plasticity material model using the 2D inverse method based on quasi-static displacements. Tam et al. [18] chose dynamic response data after Fourier transform as the objective variables to identify composite structural parameters.

As the constituent properties of textile composites are complex [27,28,29,30,31], the gradient relationship of the structural response with respect to the microscopic parameters [32,33] is difficult to determine. The complex weaving types of braided composite material together with orthotropic fiber components increase the calculation difficulty [34]. Comellas [10] proposed a modeling method for composites based on the mixing theory. The model is used to estimate the constituent isotropic parameters with the genetic algorithm. Mishra proposed a binary model to simplify the fiber-reinforced plastics model for identifying a constituent elastic modulus and structural boundary stiffness [12,14]. The binary model is mainly applied in a polymer composite, aiming at isotropic fiber material. With the development of experimental techniques in recent years [35], the heterogeneous deformation fields of composite structural tests can provide more material information during parameters’ identification; it can estimate more material parameters, for example, the constituent parameters [36]. This paper is focused on the analytical method to calculate sensitivity based on the finite element method, transforming the macroscopic and microscopic properties of the braided composite material by displacement continuity conditions.

Many attempts have already been made to deal with parameter identification based on sensitivity analysis. The sensitivity matrix can be calculated in several ways, such as the finite difference method [37,38], which has a simple formulation that can be directly applied in various analysis conditions; computations of structural response with small variations of the parameters are often required. The adjoint variable method is implemented in finite element code as a black box [39]. The substructure method of complex structures in the dynamic analysis is applied to improve computational efficiency [40]. The derivation formula of the mechanical relationship between the structural response and the parameter is relatively complex [26,41]. During parameter identification by sensitivity calculation, the ill-posed problem may result in unreliable solutions for the factors of experimental errors, data interpolation, etc. Nakamura [11] and Gras [34] converted the unknown parameters to the specific value of a modulus to decrease the magnitude differences of the parameters. Rahmani et al. identified the constituent mechanical properties with an improved regularized model updating (RMU), which reduces the influence of measured noise simultaneously [22]. Other methods such as the Kalman filter, regularized virtual fields Levenberg–Marquardt, and direct inverse maps [42] were proposed to identify parameters of composite material. Images were also used to predict the mechanical properties of materials [43].

In this paper, a sensitivity-based inverse method is proposed to identify constituent parameters of a composite; the objective function is the minimization of the discrepancies between the numerical and experimental displacement responses. The novelty of the approach is the integration of the sensitivity formula derivation with respect to constituent parameters and the parameter selection procedure. The relative sensitivity method and condition number-based response selection were applied to obtain equations with anti-noise performance for identification. The sensitivity analysis of displacement response with respect to constituent parameters is introduced in Section 2. The inverse optimization method integrated with the influence factors during identification is described in Section 3. The proposed algorithm was verified by comparing with the finite difference method using the representative volume element (RVE) of a 2.5D braided composite. The influences of identification error were discussed as well.

## 2. Sensitivity Analysis

To obtain the sensitivity matrix of the structural response with respect to the constituent parameters, the quantitative relationship of the mechanical property between the macroscopic composite and each component should be ascertained with priority, which is a complex task, especially for a braided composite. Although the sensitivity matrix can be obtained by the finite difference method directly, the computational efficiency is a crucial problem because of the requirement of repeated structural analysis at each sensitivity calculation step. A sensitivity analysis method of a structural displacement response directly with respect to the constituent parameters is proposed in this section; it can be applied to identify mesoscopic properties of the composite without knowing the relationship between the macroscopic and microscopic properties of the composite.

### 2.1. Constitutive Material Model

The basic theory of sensitivity analysis is derived from the foundation of the braided composite constitutive model, which can be characterized by the microscopic parameters. According to the finite element governing equation based on the constitutive model [44], the relationship between nodal force ***f*** and nodal displacement ***δ*** of the *e*-th element is expressed as

(1) $${\left\{f\right\}}^{e}={\left[k\right]}^{e}{\left\{\delta \right\}}^{e}$$

where ***k*** is the element stiffness matrix. Assuming that element *e* has ***l*** degrees of freedom, ***f_i_*** and ***δ_i_*** are arrays sized ***l*** × 1. The element can be expressed

(2) $${\left[k\right]}^{e}={\left[\begin{array}{cccc} k_{11} & k_{12} & \cdots & k_{1l}\\ k_{21} & k_{22} & \cdots & k_{2l}\\ \vdots & \vdots & \ddots & \vdots \\ k_{l1} & k_{l2} & \cdots & k_{ll} \end{array}\right]}^{e}$$

where ***k_ij_*** (***i***, ***j*** = 1~***l***) is the matrix element, which is related to the material parameters and the nodal location. For composite structures in a macroscopic scale, the relationship between external load ***P*** and structural displacements ***u*** is

(3) $$\left[Κ\right]\left\{u\right\}=\left[P\right]$$

The above equation can be superposed by the corresponding relationship of nodes in elements, which connect the macroscopic and mesoscopic properties. The superposition results of each element’s nodal force and nodal displacement are, respectively, the external loads and the structural displacements [17].

In addition to the corresponding nodes, the unification of elements’ material direction is necessary during the superposition period. Taking one representative volume element (RVE) of a composite as an objective, the local material coordinate systems of the braided composite are established along the axis direction of the fiber.

**Figure 1:**
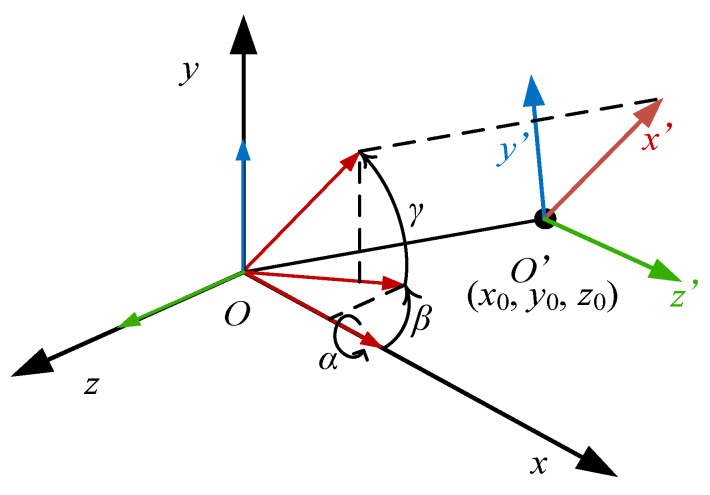
Transformation of material coordinate system.

There are two coordinate transformations, including rotation and translation, as shown in Figure 1. Each local coordinate system *O*′*-x*′*y*′*z*′ can be regarded as transforming from global coordinate system *O-xyz* with two steps.

Supposing that two systems have the same origin, the local system is obtained by the global coordinate system rotating around the *x*-axis, *y*-axis, and *z*-axis, respectively. In a right-hand coordinate system, the transformation matrix from local coordinate to global coordinate can be expressed as

(4) $$\left[T\right]=\left[\begin{array}{ccc} 1 & 0 & 0\\ 0 & \cos\alpha & -\sin\alpha \\ 0 & \sin\alpha & \cos\alpha \end{array}\right]\left[\begin{array}{ccc} \cos\beta & 0 & \sin\beta \\ 0 & 1 & 0\\ -\sin\beta & 0 & \cos\beta \end{array}\right]\left[\begin{array}{ccc} \cos\gamma & -\sin\gamma & 0\\ \sin\gamma & \cos\gamma & 0\\ 0 & 0 & 1 \end{array}\right]$$

in which *α*, *β*, and *γ* are the rotating angles around the *x*-axis, *y*-axis, and *z*-axis, respectively. The coordinate of *O*′ in the global coordinate system (*x*_0_, *y*_0_, *z*_0_) is the translation distance of the local coordinate system. Supposing ***P*** is any point in the system, the coordinate of ***P*** in the global and local system is (*x*, *y*, *z*) and (*x*′, *y*′, *x*′), respectively. The transformation relationship between the global system and the local system can be expressed as

(5) $$\left[\begin{array}{l} x\\ y\\ z\\ 1 \end{array}\right]=\left[\begin{array}{cccc} T_{11} & T_{12} & T_{13} & x_{0}\\ T_{21} & T_{22} & T_{23} & y_{0}\\ T_{31} & T_{32} & T_{33} & z_{0}\\ 0 & 0 & 0 & 1 \end{array}\right]\left[\begin{array}{l} x^{\prime }\\ y^{\prime }\\ z^{\prime }\\ 1 \end{array}\right]$$

To transfer the stiffness matrix from a local coordinate to a global coordinate, the *e^th^* element’s stiffness matrix in a global coordinate is

(6) $${\left[k\right]}_{G}^{e}={\left[T\right]}^{eT}{\left[k\right]}_{L}^{e}{\left[T\right]}^{e}$$

where the subscripts *G* and *L* represent the structure parameters in the global and local system, respectively. ***T*** is the matrix for transforming the local coordinate system to global ones.

The linear elastic constitutive equation satisfies Hooke’s law. Considering every single element, each element satisfies. Combined with the finite element theory, the relationship of Cauchy stress and nodal displacement can be equal, as

(7) $${\left\{\sigma \right\}}^{e}={\left[D\right]}^{e}{\left\{\varepsilon \right\}}^{e}={\left[D\right]}^{e}{\left[B\right]}^{e}{\left\{\delta \right\}}^{e}$$

In the above equation, ***D*** is the elastic coefficient matrix, ***B*** is the matrix-related strains resulting from the differentiating operator on the shape function ***N***, and ***δ*** is the matrix-related stresses equaling the multiplication of ***D*** and ***B***. According to the virtual work principle, the stiffness matrix of an element can be expressed as

(8) $${\left[k\right]}^{e}=\int_{V^{e}}{\left[B\right]}^{eT}{\left[T\right]}^{eT}{\left[D\right]}^{e}{\left[T\right]}^{e}{\left[B\right]}^{e}dV$$

in which *V^e^* is the volume of the *e*th element. The above equation is universal for all the solutions of an element stiffness matrix in the finite element displacement method. As shown above, the structural stiffness matrix is superposed through the nodes’ correspondence between elements. If the elements’ type and property of the homogeneous structure are identical, the structural stiffness matrix can be calculated as

(9) $$\left[K\right]=\sum_{i=1}^{N}{\left[G\right]}^{iT}{\left[k\right]}^{i}{\left[G\right]}^{i}$$

in which *N* is the total number of structural elements and ***G*** is the conversion matrix between the structure and element degree of freedom of nodes, which, similarly, can represent the structural loads from an element force as

(10) $$\left\{P\right\}=\sum_{i=1}^{N}{\left[G\right]}^{iT}{\left\{f\right\}}^{i}$$

The structural stiffness matrix of a heterogeneous structure is assembled from the constituent materials’ stiffness matrix:

(11) $$\left[K\right]=\sum_{c=1}^{n}\left(\sum_{i=1}^{M_{c}}{\left[G\right]}_{c}^{iT}{\left[k\right]}_{c}^{i}{\left[G\right]}_{c}^{i}\right)=\sum_{c=1}^{n}{\left[K\right]}_{c}$$

where *n* is the total number of the components in the structure and *M* is the element number of each component

(12) $$N=\sum_{c=1}^{n}M_{c}$$

The braided composite material is microscopically heterogeneous and is composed of an isotropic matrix and transversely isotropic fiber constituent. The different braiding angles for both the fiber and matrix inevitably cause the difference between the composite structural direction and the components’ element direction. It is worth stressing that each split of the elemental stiffness matrix is transferred to the global coordinate prior to ensuring the structural stiffness matrix under the global coordinate.

### 2.2. Analytical Derivation

Choosing elastic displacements as objective variables to identify constituent material parameters, the sensitivity matrix can be calculated by differentiating the system of structural displacements, which is derivable from Equations (6)–(12),

(13) $$\left\{u\right\}={\left(\sum_{c=1}^{n}\left(\sum_{i=1}^{M}{\left[G\right]}_{c}^{iT}{\left[k\right]}_{c}^{i}{\left[G\right]}_{c}^{i}\right)\right)}^{-1}\left\{P\right\}$$

While the nodal forces’ vector is independent of the material parameters, the partial derivative result of the component parameter *p_j_* (*j* ∈ N^+^, *j ≤ m*) into Equation (13) is

(14) $$\frac{\partial \left\{u\right\}}{\partial p_{j}}=-{\left(\sum_{c=1}^{n}{\left[K\right]}_{c}\right)}^{-2}\frac{\partial {\left[K\right]}_{j}}{\partial p_{j}}\left\{P\right\}=-{\left(\sum_{c=1}^{n}{\left[K\right]}_{c}\right)}^{-1}\frac{\partial {\left[K\right]}_{j}}{\partial p_{j}}\left\{u\right\}$$

where ***K_j_*** is the component structural stiffness matrix corresponding to the constituent’s parameter *p_j_*. The derivative of ***K****_j_* to *p_j_* can be expressed by Equation (9)

(15) $$\frac{\partial {\left[K\right]}_{j}}{\partial p_{j}}=\sum_{i=1}^{M_{j}}{\left[G\right]}_{j}^{iT}\frac{{\left[k\right]}_{j}^{i}}{\partial p_{j}}{\left[G\right]}_{j}^{i}$$

Combining Equation (15) with Equation (8), one can obtain:

(16) $$\frac{{\left[k\right]}_{j}^{e}}{\partial p_{j}}=\int_{V^{e}}{\left[B\right]}^{T}{\left[T\right]}^{T}\frac{\partial {\left[D\right]}_{j}}{\partial p_{j}}\left[T\right]\left[B\right]dV$$

Calculating the derivatives of displacements’ response with respect to constituent parameters, the sensitivity matrix ***S*** is

(17) $$\left[S\right]=\left[\begin{array}{cccc} \frac{\partial u_{1}}{\partial p_{1}} & \frac{\partial u_{1}}{\partial p_{2}} & \cdots & \frac{\partial u_{1}}{\partial p_{m}}\\ \frac{\partial u_{2}}{\partial p_{1}} & \frac{\partial u_{2}}{\partial p_{2}} & \cdots & \frac{\partial u_{2}}{\partial p_{m}}\\ \vdots & \vdots & \ddots & \vdots \\ \frac{\partial u_{s}}{\partial p_{1}} & \frac{\partial u_{s}}{\partial p_{2}} & \cdots & \frac{\partial u_{s}}{\partial p_{m}} \end{array}\right]$$

in which *s* is the total number of experimental measuring points and *m* is the total number of unknown constituent parameters.

It is clear from the above equations that the sensitivity matrix for a constituent of the braided composite proposed is in the promise of the element type and the characteristics of the composite structure. The transformation between the macroscopic and microscopic material constitutive relation can be skipped over. Other objective variables can use a similar derivation method to obtain the sensitivity matrix of response for constituent parameters.

## 3. Parameter Identification Algorithm

### 3.1. Inverse Method

The proposed inverse method for unknown parameters’ identification can be divided into the following steps. Firstly, assuming initial values, the given initial parameters of the constitutive tensor are used to construct the model of each component for estimating the displacement response of the braided composite. Secondly, identify unknown parameters iteratively by decreasing the discrepancies between the calculated response and the experimental response.

The material parameter identification is implemented by minimizing the objective function iteratively, which can be defined as the discrepancy between the experimentally measured and the numerically computed responses. Thus, the objective function can be expressed as a least-squares formulation:

(18) $$J(p)=\mathrm{Min}\sqrt{\sum_{i=1}^{s}{\left(y_{i}^{\exp}(p)-y_{i}^{\mathrm{num}}(p)\right)}^{2}}$$

where *y*^num^ and *y*^exp^ are the numerically computed and the experimentally measured responses, respectively and ***p*** is the vector of parameters to be identified.

The basic equation relating the changes in responses and the difference in material parameters can be represented by a Taylor expansion as

(19) $$y_{i}^{k+1}\approx y_{i}^{k}+\sum_{j=1}^{m}S_{ij}^{k}(p_{j}^{k+1}-p_{j}^{k})$$

where *y^k^* is the numerically computed response at step *k*.

The identifying parameters at iteration step *k* are calculated from the equation given by

(20) $${\left\{\Delta p\right\}}^{k+1}={\left({\left[S\right]}^{kT}{\left[S\right]}^{k}\right)}^{-1}{\left[S\right]}^{kT}\left({\left\{y\right\}}^{\exp}-{\left\{y\right\}}^{\mathrm{num}}\right)$$

where Δ***p*** is the identified increment of parameters at each iteration step. Both the sensitivity matrix and the numeric response should be reanalyzed at each iteration step. The convergence criterion is defined as follows:

(21) $$\sum_{i=1}^{i=s}\frac{\left|p_{i}^{k}-p_{i}^{\exp}\right|}{p_{i}^{\exp}}<\varepsilon$$

in which *ε* is the given upper bound considering the accuracy requirement during identification.

**Figure 2:**
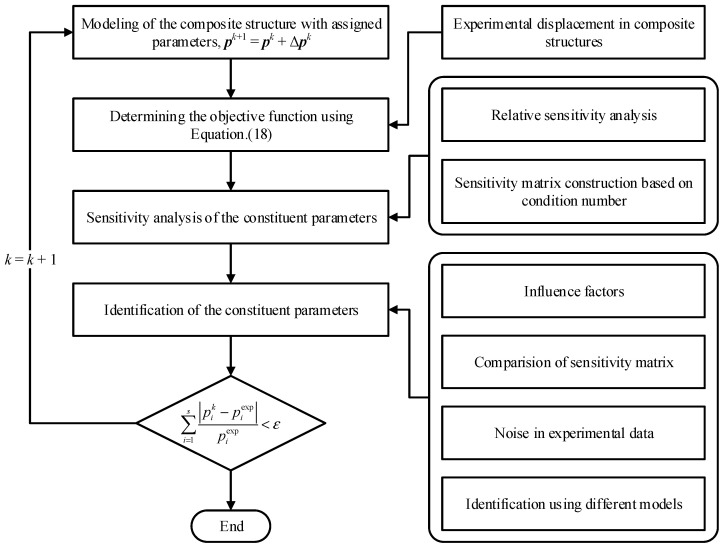
Flowchart of the constituent parameter identification method for composites.

Figure 2 shows the scheme of the proposed inverse method for identifying material parameters. Firstly, construct the finite element model and complete the structural analysis based on the assigned initial values and conduct an experimental study for obtaining data of displacement. Secondly, combining the sensitivity analysis result with the influence factors may affect the result of identification; the formula of parameters’ iteration updating in the inverse method is given. Whether the iteration ends or not depends on the convergence criterion.

### 3.2. Influence Factors

Substituting the sensitivity matrix into Equation (20) can theoretically realize the inverse method for identifying the unknown parameters. However, the ill-posed problem is unavoidable during the optimization procedure. For example, the order of magnitude of a composite elastic modulus and shear modulus are usually 9 and 6, while the Poisson’s ratio is 1, resulting in the magnitude differences of the sensitivity corresponding to each variable. Premature local convergence during the identification process will severely affecting the identification precision and efficiency. The modulus and Poisson’s ratios being identified for the constituent parameters’ identification of composite structure are a typical ill-posed problem. A common method to solve this problem is to replace the identified parameters with the specific of two unknown parameters with similar magnitude. However, this method requires calculating the sensitivity again. Moreover, the new sensitivity analysis may bring in new ill-posed problems during the identification process. The identification method used for this paper selected the relative sensitivity to solve the influence of magnitude differences among parameters.

The principle of the relative sensitivity method is substituting the optimization equation after dimensionless treatment. Considering the definition of the sensitivity matrix, the relative sensitivity matrix can be expressed as

(22) $$S_{ij}^{\prime }=\frac{1}{u_{i}}\cdot S_{ij}\cdot p_{j}=\frac{1}{u_{i}}\cdot \frac{\partial u_{i}}{\partial p_{j}}\cdot p_{j}$$

where $S_{ij}^{\prime }$ and *S_ij_* represent the element of relative sensitivity and the sensitivity matrix, respectively (*i* = 1, 2,…, *s*; *j* = 1, 2,…, *m*). The increment parameters in the *k*th step can be transformed as

(23) $$\left\{\begin{array}{l} {\left\{\delta \right\}}^{k+1}={\left({\left[S^{\prime }\right]}^{kT}{\left[S^{\prime }\right]}^{k}\right)}^{-1}{\left[S^{\prime }\right]}^{kT}\left(\frac{{\left\{y\right\}}_{\exp}-{\left\{y\right\}}_{\mathrm{num}}^{k}}{{\left\{y\right\}}_{\mathrm{num}}^{k}}\right)\\ {\left\{\Delta p\right\}}^{k+1}={\left\{p\right\}}^{k}\times {\left\{\delta \right\}}^{k+1} \end{array}\right.$$

where the updated convergence criteria are

(24) $$\sum_{i=1}^{i=s}\left|\delta^{k}\right|<\varepsilon$$

In addition to magnitude difference, choosing objective variables can influence the identified result. Take the displacement as an example. The application of the full-field measurement method is mature enough to satisfy the measurement requirements. A proper quantity of measuring points and choosing the objective measuring direction are required for the identified efficiency. Furthermore, the measuring errors during the experiment would influence the accuracy of parameters’ identification, which varies with the number of measuring points. To decrease the ill-posed problem caused by measuring points, the condition number of the sensitivity matrix is proposed to determine the measuring numbers, choosing

(25) $$C(S)=\left\|S\right\|\cdot \left\|S^{-1}\right\|$$

The condition number is a measure of the degree of ill condition for the matrix. The smaller the condition number is, the better the identified results are. The condition number does not have linear variation varying with the measuring points. The condition number of the sensitivity matrix is an important indicator in determining the amount of measuring points.

## 4. Numerical Simulated Examples and Discussion

**Figure 3:**
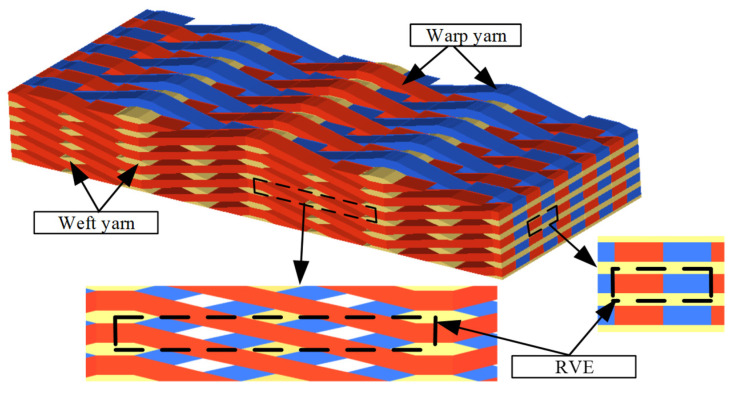
Geometry of 2.5D braided composite.

To verify the identification method for composite constituent parameters, a 2.5D braided composite was proposed for this paper. The textiles are usually braided or knitted by the yarns, bundled from thousands of fibers. In particular, the 2.5-dimension composite utilizes fiber preforms constructed from straight and sinusoidal yarns arranged into complex 2.5D structures. The weaving fabric architecture of a fiber preform is shown in Figure 3, which includes the straight weft yarns and sinusoidal warp yarns in two vertical directions, with the adjacent layers of weft yarns interlaced together by warp yarns. The sinusoidal warp yarns interlock the warp yarns, while all the adjacent weft yarns are in the same situation in the straight weft direction.

**Figure 4:**
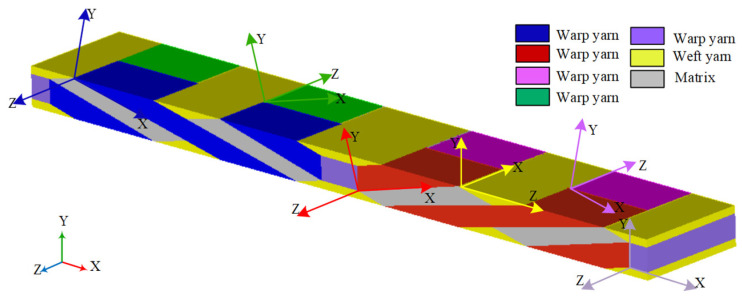
Material coordinate systems of the RVE of braided composite.

The dimensions of the geometrical RVE model are 12.9 mm × 2 mm × 0.66 mm, while the volume fraction of the fiber preform is 40%. Table 1 shows the geometrical parameters of the weave architecture of the composite. The yarns composited of fiber and matrix are treated as unidirectional fiber-reinforced composite materials during identification. The natural coordinate system of fiber alternates along with the path of the fiber. The schematic of the local coordinate system based on different weaving directions in the RVE is shown in Figure 4, where the RVE is divided into six parts. The constituent properties assumed are presented in Table 2, where fiber and matrix are transverse isotropic and isotropic material, respectively [43].

Numerical examples were attempted for two distinct cases: a sensitivity matrix comparison with finite difference method and influence factors discussion during identification, especially with measuring noise. The solid model was proposed in a later case to fulfill the simulation.

**Case** **1.** *Sensitivity matrix comparison*

The 2.5D RVE model was considered in this case study. Calculating the sensitivity matrix through the direct derivation and finite difference methods, the inverse method coupling with relative sensitivity analysis was proposed to identify the constituent parameters of a braided composite. The finite forward difference was used [13]

(26) $$S_{ij}^{FD}=\frac{u_{i}\left(p+\Delta p_{j}\right)-u_{i}\left(p\right)}{\Delta p_{j}}$$

in which Δ*p_j_* is the relative perturbation on *j*th parameter, whose selection remains to be a controversial topic as the truncation error and round-off error of sensitivity calculated above varied with the perturbation. Considering the output accuracy of the response in the software, the perturbation value in the present study was set to Δ*p_j_* = 0.01 × *p_j_.* The upper-bound error *ε* of the convergence criterion given here was 5 × 10^−3^.

**Figure 5:**
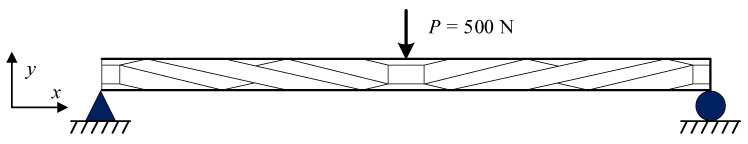
Three-point bending test diagram of the RVE.

Figure 5 shows the schematic diagram for the 2.5D braided RVE model. Applying a three-point bending test on the RVE, the PATRAN/NASTRAN software was used to complete the forward method, obtaining the structure displacements and objective variables.

Two group material parameters far from true values were chosen as initial values for identification. The accuracy and the efficiency of the two methods were discussed in the following case. The influence of measuring points during identification was discussed; we determined that too many measuring points would increase the number of iterations. The simulation here extracted six points of displacements of the x and y direction as the objective variables.

Table 3 shows the identification result and errors with six measuring points by two sensitivity calculation methods, respectively. The identified errors were ratios resulting from the discrepancy between identified and true parameters. From the table, we can see that both methods correctly identified the constituent mechanical properties of the 2.5D braided composite and the identified errors shared similar rules, where the identified errors of the elastic modulus were smaller than that of Poisson’s ratios. Therefore, the determined result of the fiber of Poisson’s ratio in the transverse direction had a large error over 5%. Indeed, the objective of the relativity method in the constituent parameters’ identification can effectively solve a potential problem to some extent. Few measuring points during identification may cause a large number of identified errors or other problems; so, it is important to find a proper quantity of objective variables before identification.

**Figure 6:**
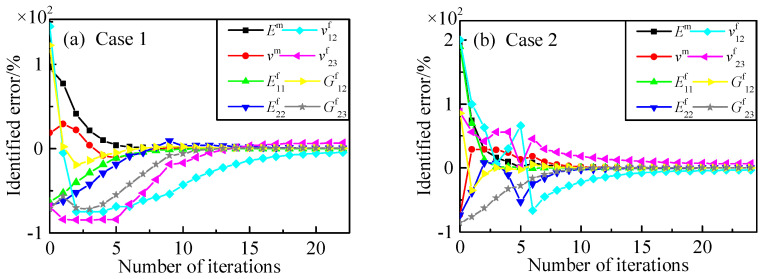
Identified process for constituent parameters with six points (the direct derivation method). (**a**) initial values: *E*^m^ = 8 GPa, *ν*^m^ = 0.45, E^f^_11_ = 100 GPa, E^f^_22_ = 6 GPa, *ν*^f^_12_ = 0.49, *ν*^f^_23_ = 0.1, G^f^_12_ = 60 GPa, G^f^_23_ = 2 GPa; (**b**) initial values: *E*^m^ = 12 GPa, *ν*^m^ = 0.1, E^f^_11_ = 800 GPa, E^f^_22_ = 5 GPa, *ν*^f^_12_ = 0.6, *ν*^f^_23_ = 0.6, G^f^_12_ = 50 GPa, G^f^_23_ = 1 GPa.

**Figure 7:**
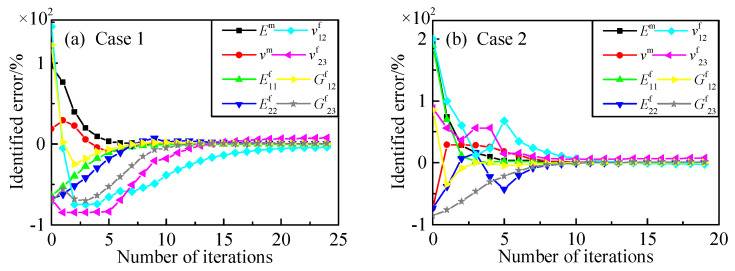
Identified process for constituent parameters with six points (the finite difference method). (**a**) initial values: *E*^m^ = 8 GPa, *ν*^m^ = 0.45, E^f^_11_ = 100 GPa, E^f^_22_ = 6 GPa, *ν*^f^_12_ = 0.49, *ν*^f^_23_ = 0.1, G^f^_12_ = 60 GPa, G^f^_23_ = 2 GPa; (**b**) initial values: *E*^m^ = 12 GPa, *ν*^m^ = 0.1, E^f^_11_ = 800 GPa, E^f^_22_ = 5 GPa, *ν*^f^_12_ = 0.6, *ν*^f^_23_ = 0.6, G^f^_12_ = 50 GPa, G^f^_23_ = 1 GPa.

Figure 6 and Figure 7 show the identification process for the constituents of the 2.5D braided composite with six measuring points. The direct derivation method and the finite difference method shared similar convergence rules during identification, while the latter method required more iterative steps than the former one. Furthermore, the calculation principle of the finite difference method required eight times forward analysis at each iterative step, while the finite element method can accomplish sensitivity analysis by one forward calculation. The sensitivity calculated by the direct derivation method had higher calculation efficiency.

Keep other conditions unchanged and add one measuring point during identification. From the results shown in Table 4, we can see the identified errors of the direct derivation method decreased with the addition of the measuring points while the Poisson’s ratio of fiber in the transverse direction distorted with the finite difference method.

**Figure 8:**
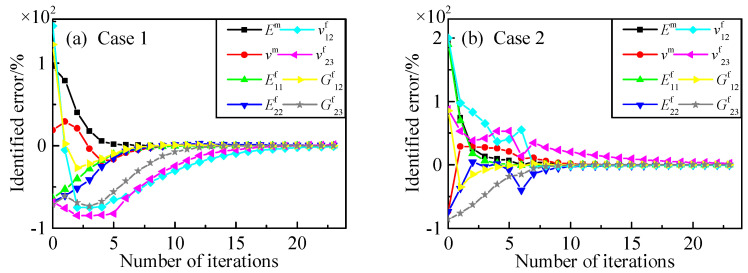
Constituent parameter identification with seven points using direct derivation. (**a**) initial values: *E*^m^ = 8 GPa, *ν*^m^ = 0.45, E^f^_11_ = 100 GPa, E^f^_22_ = 6 GPa, *ν*^f^_12_ = 0.49, *ν*^f^_23_ = 0.1, G^f^_12_ = 60 GPa, G^f^_23_ = 2 GPa; (**b**) initial values: *E*^m^ = 12 GPa, *ν*^m^ = 0.1, E^f^_11_ = 800 GPa, E^f^_22_ = 5 GPa, *ν*^f^_12_ = 0.6, *ν*^f^_23_ = 0.6, G^f^_12_ = 50 GPa, G^f^_23_ = 1 GPa.

**Figure 9:**
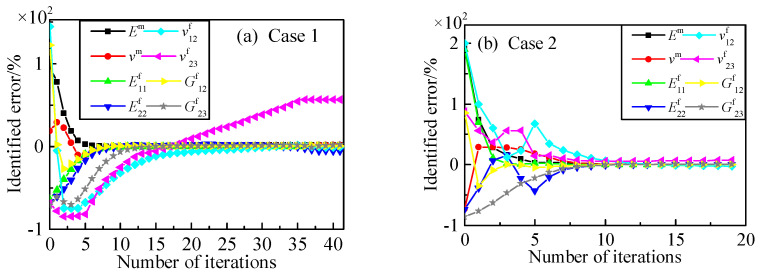
Constituent parameter identification with seven points using finite difference. (**a**) initial values: *E*^m^ = 8 GPa, *ν*^m^ = 0.45, E^f^_11_ = 100 GPa, E^f^_22_ = 6 GPa, *ν*^f^_12_ = 0.49, *ν*^f^_23_ = 0.1, G^f^_12_ = 60 GPa, G^f^_23_ = 2 GPa; (**b**) initial values: *E*^m^ = 12 GPa, *ν*^m^ = 0.1, E^f^_11_ = 800 GPa, E^f^_22_ = 5 GPa, *ν*^f^_12_ = 0.6, *ν*^f^_23_ = 0.6, G^f^_12_ = 50 GPa, G^f^_23_ = 1 GPa.

Figure 8 shows the parameter identification process using the direct derivation method. The identification process is quite similar to the process with six measuring points. The constituent parameters’ identification based on the direct derivation method sensitivity analysis can obtain better identified precision with the increase in a referenced response. Comparing Figure 9 with Figure 7, the biggest difference between the two identification processes is the identified plot of *v*^f^_23_, where the result of sensitivity bias of Poisson’s ratio, calculating from finite difference, is presented; the chosen relative perturbation for finite differences may influence sensitivity accuracy, especially with some parameters having nonlinearity variation rules. Comparing with other parameters, *v*^f^_23_ varied flexibly with the displacements’ response of a three-point bending test, while the response was less sensitive to *v*^f^_23_, which resulted in the error on parameter identification, shown in Figure 9. When the objective function was close to zero, the sensitivity of *v*^f^_23_ faced a new ill-posed problem, which could not be eliminated simply by the relative sensitivity method. The perturbation chosen could influence the identification accuracy as there exists a different extent of sensitivity variation in respect to different parameters. According to the comparison between the direct derivation method and finite difference method, the verification of the sensitivity formula derivation and the relative sensitivity method applying to microscopic parameters’ identification was accomplished.

**Case** **2.** *Influences factors with measuring noise*

As shown in case 1, the quantity of measuring points may influence the identification result of the composite. The following cases’ analysis identification resulted with the increase in measuring points, considering measuring noise during identification. A 0.5% unbiased white Gaussian noise was proposed to respond as the measuring noise. The extracted displacements in x and y directions were chosen as the objective variables firstly.

**Figure 10:**
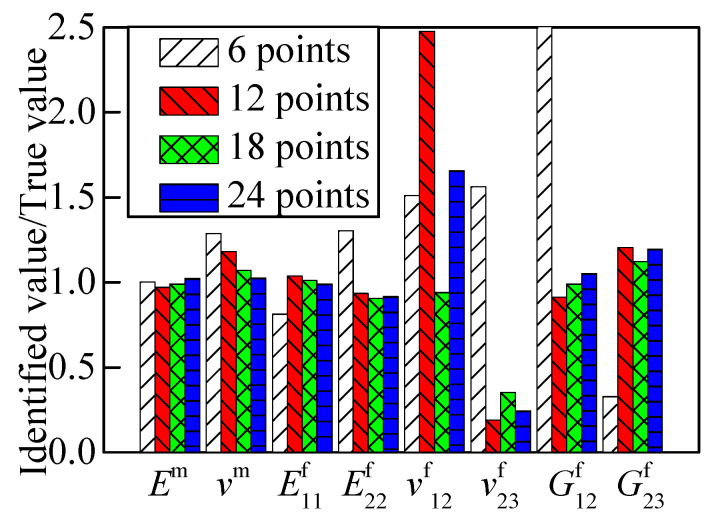
RVE identified result with different numbers of points (x and y directions).

**Figure 11:**
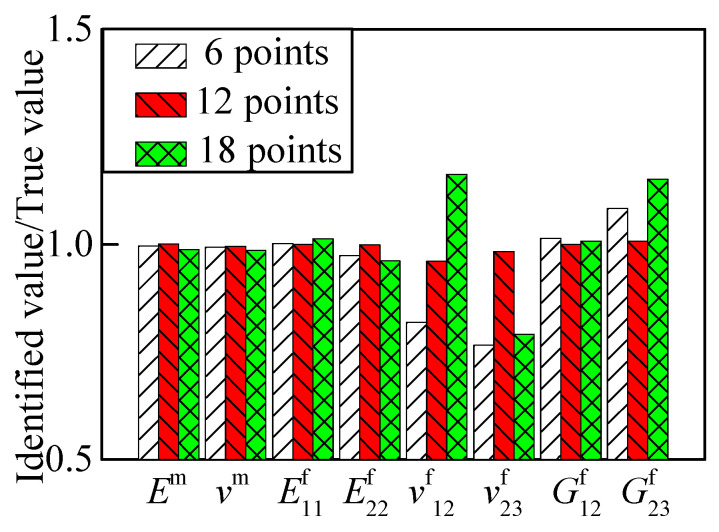
RVE identified result with different numbers of points (three directions).

Table 5 shows the identified errors of a 2.5D braided composite material varying with the quantity of measuring points. None of the chosen cases satisfied the convergence criterion after 30 times of iteration. Observing the value ratios in Figure 10, the convergence of *v*^f^_12_, *v*^f^_23,_ and *G*^f^_23_ had relatively high errors. Abstracting three directions of displacement response, the identified results are shown in Figure 11. The identified results with three displacement directions tended towards stability, and the case with 12 measuring points obtained a satisfactory identification result.

**Figure 12:**
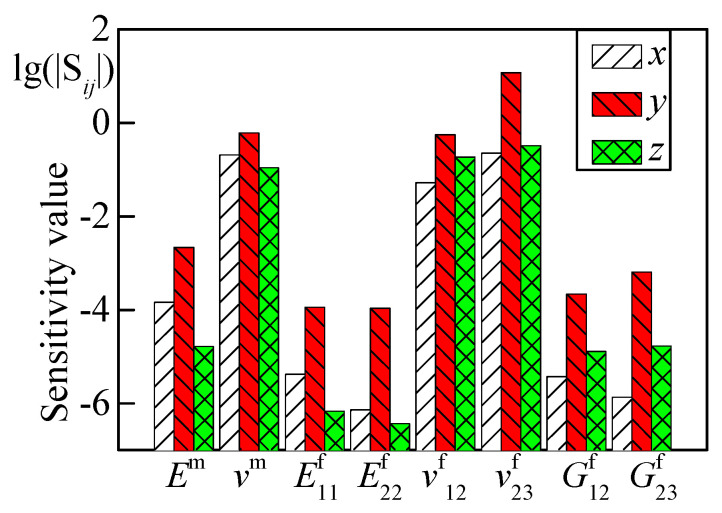
Sensitivity for displacements in respect to constituent parameters.

The Poisson’s ratio of fiber and shear modulus in 23 directions had a local convergence problem when adding white Gaussian noise to objective variables. We chose one measuring node to observe its sensitivity of static displacements with respect to each parameter, as shown in Figure 12. The Figure 12 shows that the sensitivity for displacements with respect to Poisson’s ratio was much higher than that with respect to modulus, which is one reason for a relative sensitivity matrix.

The condition number of the relative sensitivity matrix in different cases is shown in Table 6. The lower the condition number the relative sensitivity matrix had, the more accuracy the identified result had. On the other hand, the 2.5D RVE model was asymmetric. Adding white Gaussian noise could influence some identification results in fiber only by x and y direction measuring data.

**Case** **3.** *Estimation parameters using a panel model*

Constructing a 2.5D braided composite model based on the transformation of an RVE model, three-point bending was applied in the model in the y direction as the schematic diagram for the RVE model. The size of the solid model was 38.7 mm × 8 mm × 2.64 mm. The eight elastic parameters of the fiber and matrix were identified considering the measuring error. Determination of the measuring points’ amount was based on the condition number of relative sensitivity. According to the calculated condition number shown in Table 7, we selected 29 points in two-direction measurement and 18 points in three-direction measurement, respectively. The initial value was the same as that in case 1. The white Gaussian noise with 0.5% was added to the solid model. Identification results of different measuring directions are shown in Table 8. The fiber’s transverse parameters had a local convergence problem measuring in two directions, while all constituent parameters were successfully identified measuring in three directions. The results demonstrated the certification method of the response on identification to the influence of measuring noise.

**Figure 13:**
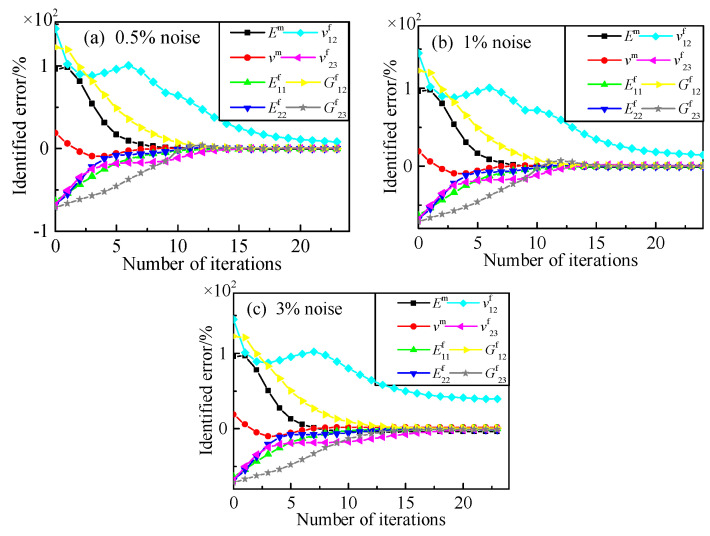
Identified process for constituent parameters of composite plane. (**a**) 0.5% noise; (**b**) 1% noise; (**c**) 3% noise.

We added white Gaussian noise with 1% and 3% to objective variables, respectively. Table 9 and Figure 13 show the identification results. From the result data, we can see the identified error of *v*^f^_12_ increased with measuring noise. However, the other seven constituent parameters had a relatively high identification accuracy. This is basically because of the determination of the convergence criterion.

## 5. Conclusions

A constituent parameter identification method was proposed based on a sensitivity analysis of structural displacements. Identification effect factors such as the relative sensitivity analysis and response point selection were investigated. Simulation studies on identifying the elastic parameters were conducted by employing a 2.5-dimensional braided composite; conclusions on the proposed method were drawn as follows.

1. The sensitivity analysis of displacement with respect to parameters of constituents was derived. Coupling the relative sensitivity and condition number-based experimental point selection, the noise-polluted problem during identification could be solved. It was shown that the proposed sensitivity method was more effective and accurate compared with the finite difference method.
2. The amount of measuring points greatly influenced the identification accuracy when there were existing noises in the measurement. For the diversity of constituent parameters and the structural complexity of the braided composite, the condition number of the relative sensitivity matrix could be a reliable index for response selection.
3. The identification result will be influenced by the selection of structural responses. Prior information of the initial parameters and the value range can effectively improve identification efficiency.

## Figures and Tables

**Table 1:** Geometrical parameters of weave architecture in 2.5D RVE.

Warp Density/(Ends/cm)	Rectangular Warp Yarn		Elliptical Weft Yarn		Parallelogram Weft Yarn	Crimp Angle of Warp Yarns/deg	WEFT Density/(Picas/cm)
	Width/mm	Height/mm	Major Axis/mm	Minor Axis/mm	Length of Side/mm		
10	1	0.39	1.8	0.26	0.71	14	3

**Table 2:** Constituent properties for the braided composite.

	Modulus/GPa				Poisson’s Ratio	
	*E*_11_	*E*_22_	*G*_12_	*G*_23_	*ν*_12_	*ν*_23_
Fiber	276	19	27	7	0.2	0.32
Matrix	4.08		1.48		0.38	

**Table 3:** Identification result for two sensitivity methods (six points).

Case	Parameter	Initial Data	Proposed Sensitivity Method		Finite Difference Method	
			Identified Data	Identified Error %	Identified Data	Identified Error %
1	*E*^m^/GPa	8	4.089	0.22	4.091	0.27
	*ν* ^m^	0.45	0.381	0.26	0.381	0.26
	*E*^f^_11_/GPa	100	275.991	−0.003	275.96	−0.014
	*E*^f^_22_/GPa	6	19.045	0.24	19.072	0.38
	*ν* ^f^_12_	0.49	0.191	−4.55	0.192	−3.99
	*ν* ^f^_23_	0.1	0.340	6.25	0.344	7.44
	*G*^f^_12_/GPa	60	27.058	0.21	27.065	0.24
	*G*^f^_23_/GPa	2	7.040	0.57	7.046	0.66
2	*E*^m^/GPa	12	4.089	0.22	4.092	0.29
	*ν* ^m^	0.1	0.381	0.26	0.381	0.26
	*E*^f^_11_/GPa	800	275.945	−0.02	275.892	−0.04
	*E*^f^_22_/GPa	5	19.019	0.10	19.079	0.42
	*ν* ^f^_12_	0.6	0.193	−3.5	0.195	−2.5
	*ν* ^f^_23_	0.6	0.341	6.56	0.344	7.55
	*G*^f^_12_/GPa	50	27.056	0.21	27.066	0.24
	*G*^f^_23_/GPa	1	7.039	0.56	7.045	0.64

**Table 4:** Identification results for two sensitivity measuring method (seven points).

Case	Parameter	Initial Data	Proposed Sensitivity Method		Finite Difference Method	
			Identified Data	Identified Error %	Identified Data	Identified Error %
1	*E*^m^/GPa	8	4.082	0.06	4.144	1.56
	*ν* ^m^	0.45	0.380	0.05	0.387	1.75
	*E*^f^_11_/GPa	100	276.040	0.01	275.777	−0.08
	*E*^f^_22_/GPa	6	19.030	0.16	17.958	−5.49
	*ν* ^f^_12_	0.49	0.197	−1.25	0.198	−0.92
	*ν* ^f^_23_	0.1	0.322	0.73	0.500	56.25
	*G*^f^_12_/GPa	60	27.006	0.02	27.143	0.53
	*G*^f^_23_/GPa	2	7.004	0.06	7.130	1.86
2	*E*^m^/GPa	12	4.082	0.06	4.143	1.54
	*ν* ^m^	0.1	0.380	0.11	0.386	1.67
	*E*^f^_11_/GPa	800	275.992	0.003	275.745	−0.09
	*E*^f^_22_/GPa	5	18.951	−0.26	17.969	−5.43
	*ν* ^f^_12_	0.6	0.200	−0.21	0.199	−0.65
	*ν* ^f^_23_	0.6	0.328	2.52	0.499	56.25
	*G*^f^_12_/GPa	50	27.005	0.02	27.147	0.54
	*G*^f^_23_/GPa	1	7.006	0.09	7.122	1.75

**Table 5:** Identified errors varying with amount of measuring points.

Direction	Points	Identified Errors %							
		*E* ^m^	*ν* ^m^	*E* ^f^_11_	*E* ^f^_22_	*ν* ^f^_12_	*ν* ^f^_23_	*G* ^f^_12_	*G* ^f^_23_
*x*, *y*	6	0.23	28.91	−18.76	30.29	50.90	56.25	190.1	−67.24
	12	−2.99	18.28	3.86	−6.55	147.5	−81.01	−8.65	20.43
	18	−0.99	7.19	1.05	−9.54	−6.02	−64.79	−0.94	12.21
	24	2.13	2.38	−1.06	−8.25	65.75	−75.57	5.30	19.46
*x*, *y*, *z*	6	−0.28	−0.69	0.25	−2.65	−18.15	−23.48	1.36	8.36
	12	0.11	−0.45	−0.03	−0.16	−3.88	−1.67	0.05	0.81
	18	−1.18	−1.36	1.28	−3.79	16.23	−20.88	0.78	15.16

**Table 6:** Condition number of relative sensitivity matrix for RVE.

Measuring Direction	Measuring Points	Condition Number
*x*, *y*	6	3.96 × 10^5^
	12	1.96 × 10^3^
	18	3.13 × 10^2^
	24	3.49 × 10^3^
*x*, *y*, *z*	6	2.52 × 10^3^
	12	5.84 × 10^2^
	18	9.98 × 10^2^

**Table 7:** Condition number of relative sensitivity matrix for solid model.

Measuring Direction	Measuring Points	Condition Number
*x*, *y*	24	1.08 × 10^4^
	25	1.02 × 10^4^
	26	8.57 × 10^3^
	27	8.18 × 10^3^
	28	8.07 × 10^3^
	29	7.98 × 10^3^
	30	2.95 × 10^4^
*x*, *y*, *z*	15	2.58 × 10^3^
	16	2.39 × 10^3^
	17	2.38 × 10^3^
	18	1.83 × 10^3^
	19	1.84 × 10^3^
	20	3.05 × 10^3^

**Table 8:** Identified constituent parameters of composite panel (0.5% noise).

Parameter	Initial Data	Measuring in Two Directions		Measuring in Three Directions	
		Identified Data	Identified Error %	Identified Data	Identified Error %
*E*^m^/GPa	8	3.893	−4.57	4.053	−0.65
*ν* ^m^	0.45	0.365	−3.87	0.382	0.42
*E*^f^_11_/GPa	100	279.813	1.38	275.957	−0.02
*E*^f^_22_/GPa	6	18.706	−1.54	18.938	−0.32
*ν* ^f^_12_	0.49	0.183	−8.53	0.215	7.72
*ν* ^f^_23_	0.1	0.132	−58.86	0.320	0.07
*G*^f^_12_/GPa	60	26.415	−2.17	27.006	0.02
*G*^f^_23_/GPa	2	8.352	19.31	6.999	−0.01

**Table 9:** Identified constituent parameters of composite panel.

Parameter	Initial Data	1% Noise		3% Noise	
		Identified Data	Identified Error %	Identified Data	Identified Error %
*E*^m^/GPa	8	4.026	−1.30	3.918	−3.97
*ν* ^m^	0.45	0.382	0.61	0.3867	1.75
*E*^f^_11_/GPa	100	275.992	0.00	276.227	0.08
*E*^f^_22_/GPa	6	18.876	−0.65	18.723	−1.46
*ν* ^f^_12_	0.49	0.228	13.98	0.278	39.41
*ν* ^f^_23_	0.1	0.321	0.24	0.318	−0.69
*G*^f^_12_/GPa	60	27.016	0.06	27.102	0.38
*G*^f^_23_/GPa	2	6.995	−0.07	6.946	−0.78

## Data Availability

Not applicable.
